# Gentiopicroside Ameliorates Sepsis-Induced Acute Lung Injury via Inhibiting Inflammatory Response

**DOI:** 10.1155/2024/1068326

**Published:** 2024-09-05

**Authors:** Mu Hu, Hangxiang Du, Yang Xu, Yan Wang

**Affiliations:** ^1^ Department of Orthopedics Ruijin Hospital Shanghai Jiaotong University School of Medicine, Shanghai 201801, China; ^2^ Department of Critical Care Medicine Ruijin Hospital Shanghai Jiao Tong University School of Medicine, Shanghai, China

## Abstract

Sepsis is a systemic inflammatory reaction syndrome caused by infections. Acute lung injury (ALI) occurs first and most frequently in patients with sepsis. Gentiopicroside (GPS), which originates mostly from *Gentiana,* is classified as a secoiridoid glycosides. Terpenoid glycosides have various biological effects, including liver protection, blood glucose and cholesterol level management, and anti-inflammatory and antitumor effects. However, presently, the biochemical foundation and mechanism of the anti-inflammatory effects of GPS in sepsis-induced ALI have not been explained. In the present study, we established a rat model of sepsis ALI induced by cecal ligation and puncture. This enables us to observe the effects of GPS therapy, which significantly reduced the inflammatory response (TNF-*α*, IL-1*β*, and IL-6), nitrogen stress, oxidative stress, and severity of ALI at both the whole animal and molecular levels. In addition, GPS ameliorates LPS-induced ALI via regulation of inflammatory response and cell proptosis in BEAS-2B. This study provides a theoretical basis for treating sepsis-induced ALI with GPS.

## 1. Introduction

The clinical management of severe sepsis and septic shock faces the greatest challenge, as sepsis has high mortality and incidence rates, and sepsis is regarded as the leading cause of death for patients admitted in critical care units [1]. Sepsis is a common complication after major surgeries, serious burns, ischemia and reperfusion injuries, and severe traumatic injuries, in which infections are the underlying cause [2, 3]. In addition, sepsis affects millions of individuals worldwide every year, with the incidence rate expended to increase annually [4]. It is estimated that more than 10,000 persons in the United States die of severe sepsis each year [5]. Acute lung injury (ALI) is the earliest sign among all organ injuries, and the highest incidence is closely related to sepsis [6]. In severe cases of sepsis, multiple organ function injuries can occur in sequence, with the lung being the most susceptible to injury [7–9]. The etiology of the condition encompasses various facets, such as the intensification of the inflammatory reaction, impairment of the endothelial function, and the body's response to oxidative stress. These factors collectively contribute to the breakdown of the alveolar-capillary membrane and an enhancement of blood vessel permeability. Despite extensive investigation into the mechanisms by which sepsis triggers ALI, the precise sequence of pathological events remains partially understood. Currently, no specific drugs or effective treatments exist for the disease. Treatment of sepsis-related ALI is supportive, managing symptoms through respiratory, infection, fluid, and hemodynamic support to reduce inflammation, preserve organ function, and correct metabolic issues to improve survival [10]. Beyond these, new strategies such as targeted inflammatory pathway therapy, immunomodulation, and antioxidants are being researched [11]. Therefore, exploring novel medicine to guide the clinical treatment of sepsis is needed.

The hemicyclic ether terpene glycosides, of which gentiopicroside (GPS) is a member, are derived mostly from *Gentiana scabra* [12]. According to several studies, the biological effects of GPS include liver protection, inflammation reduction, blood glucose and cholesterol regulation, and tumor growth prevention [13–15]. Intraperitoneal injection of GPS can significantly reduce the serum amylase, lipase, TNF-*α*, and IL-1*β* concentrations in rats with acute pancreatitis. The benefits of GPS on reducing inflammation and protecting against oxidative stress are also observed in patients with neurological disorders [16]. GPS may block the NF-*κ*B/MAPK signal, which reduces inflammatory neuronal damage and improves LPS-induced depression-like behavior, by regulating the tryptophan degradation signal pathway [17]. In the animal model of oxygen-glucose deprivation reperfusion injury in newborn rats, GPS lowered the death rate of hippocampal neurons [18]. This reduction may be connected to the upregulation of Bcl-2 expression and suppression of Caspase-3 and Bax expressions. In addition, GPS alleviated diabetic peripheral neuropathy by modulating the PPAR-*γ*/AMPK/ACC signal [16]. However, whether GPS alleviates the inflammation caused by sepsis-induced ALI remains unclear. More research is required to determine whether GPS has a potential as a therapeutic medication for treating acute lung damage in patients with sepsis.

In the present study, we aimed to investigate the inflammatory reaction, nitric acid stress, oxidative stress, and ALI due to sepsis treated with GPS at both the whole animal and molecular levels by using an animal model of sepsis induced by cecal ligation and puncture. It offers a novel research proposition for the prevention of ALI in patients with sepsis as well as therapeutic management of the condition.

## 2. Materials and Methods

### 2.1. Establishment of Sepsis-Induced ALI Rat Model

All rats (8–12 weeks old) were purchased from Shulaibao Biotech (Wuhan, China) and maintained at constant temperature (23–25°C) with controlled light/dark cycles (12 h/12 h) at Shanghai Jiao Tong University School of Medicine. By ligating and perforating the cecum, a sepsis-associated ALI rat model was generated. To minimize pain during all procedures, 1% pentobarbital sodium (50 mg/kg, IP) was employed as an anesthetic agent. The completely sedated rats were dissected, one-third of the cecum was ligated using a suture, and then a needle was used to ligate the middle section of the cecum. At the end of the procedure, the cecum was placed back into the abdominal cavity, and the abdominal cavity was then closed layer by layer before being cleaned. After the removal of the cecum, the cecum was not ligated or punctured in the Sham rats, rather the cecum was immediately reinserted into the abdominal cavity after it had been exposed.

### 2.2. GPS Treatment in Sepsis-Associated ALI Rat Model

Sixty Sprague Dawley rats were randomly assigned to one of the following four groups, with each group comprising 15 rats: Sham, cecal ligation and puncture (CLP), CLP + GPS-25, and CLP + GPS-50 groups. In the CLP group, the tail vein was injected with various doses (25 or 50 mg/kg) of GPS (Nanjing Guangrun Biological Products, China) immediately after the surgery [19], and the medications were given at the same time every day for 3 consecutive days.

### 2.3. Cell Culture

BEAS-2B cells were incubated in DMEM with 10% FBS at a temperature of 37°C under an atmosphere of 5% CO_2_. When the BEAS-2B cells reached approximately 80% confluence, lipopolysaccharide (LPS, Sigma-Aldrich, United States) and with or without GPS for a specified time.

### 2.4. Cell Counting Kit-8 (CCK-8) Assays

CCK-8 assays were performed based on the manufacturer's instructions (Beyotime, China). The BEAS-2B cells were treated with LPS (1 *µ*g/ml) for 4 h and with or without GPS for 6 h. A microplate reader (Omega Bio-Tek, Georgia, United States) was used for determining the optical density at 450 nm.

### 2.5. Hematoxylin-Eosin (HE) Staining

After fixing the lung tissues of rats with 4% paraformaldehyde for 48 hours, washing them with distilled water, dehydrating them until they were clear, sectioning them at 4 um thickness, dewaxing them, and then rewatering them before staining with HE, pathological alterations were observed in the lung tissues by 40x magnification light microscopy.

### 2.6. Determination of NO Release

After grinding the frozen rat lung tissue at −80°C on ice, 40 microliters of the supernatant from each sample was removed and placed in new 96-well plates. Then, 40 microliters of the Griess reagent (Merck) was added to each well; then, the plates were incubated in the dark for 30 minutes. A microplate reader was used to determine the optical density at 540 nm.

### 2.7. Enzyme-Linked Immunosorbent Assay (ELISA)

After the 24-hour administration period, blood was extracted from the rat's tail, separated by centrifugation. The TNF-*α* (BMS223-4, Thermo Fisher), IL-1*β* (KAC1211, Thermo Fisher), and IL-6 (BMS213-2, Thermo Fisher) levels in serum and bronchoalveolar lavage fluid (BALF) of the rats in each group were determined by ELISA. The procedures were performed as per the manufacturer's instruction.

### 2.8. Real-Time Quantitative PCR (RT-qPCR)

Trizol lysate was added to extract total RNA from the rat lung tissue, and cDNA was synthesized using RevertAid^TM^ First-Strand cDNA Synthesis Kit according to the manufacturer's instructions. The experiment was performed three times. The change in the relative mRNA expression level of the target gene iNOS was calculated using the 2^−△△CT^ technique. The sequence of the primers was as follows: F: 5′⁃GGGAATCTTGGAGCGAGTTG⁃3′, R: 5′⁃ GTGAGGGCTTGGCGTGA⁃3′.

### 2.9. Western Blot

The lung tissue of the rats and BEAS-2B cells was ground on ice, and the protein was extracted with lysate. The amount of protein present was measured using a BCA protein quantification kit (Shanghai Dongsheng Biotechnology, Shanghai, China). After 2 hours of sealing with 5% skim milk powder, the PVDF membrane (0.22 *μ*m, Millipore ISEQ00010, United States) was incubated with a primary antibody (iNOS (1 : 1000, ab178945, Abcam), Caspase-3 (1 : 1000, ab32351, Abcam), Bcl-2 (1 : 1000, ab32124, Abcam), Bax (1 : 1000, ab32503, Abcam), p38 (1 : 1000, ab170099, Abcam), ERK (1 : 1000, ab184699, Abcam), JNK (1 : 1000, ab179461, Abcam), and p-p38 (1 : 1000, ab195049, Abcam)) at 4°C overnight. Then, the membrane was incubated with HRP-combined secondary antibody (1 : 5000, Abcam, Waltham, MA, United States). Protein bands were detected using the Prime Western Blotting Detection Reagent (Cytiva, UK). A ChemiDoc MP imaging system (Tanon 4800, Shanghai, China) was used to detect chemiluminescence, and the Image J software was used to analyze the gray value of the bands.

## 3. MPO, SOD, MDA, and GSH Levels

The rat lung tissue was homogenized, centrifuged at 4000 r/min for 15 min, and the supernatant was separated. The MPO, SOD, MDA, and GSH levels in the lung tissue samples were determined according to the kit's instructions (Elabscience, China).

### 3.1. Statistical Analysis

Statistical analysis was done using SPSS 22.0, and the measurement data were expressed as mean ± standard deviation. The single factor analysis of variance and SNK-q test were used to compare the data among the various groups. ^#^ or ^∗^*P* < 0.05 was considered statistically significant.

## 4. Results

### 4.1. GPS Alleviates Sepsis-Induced ALI in Rats

**Figure 1:**
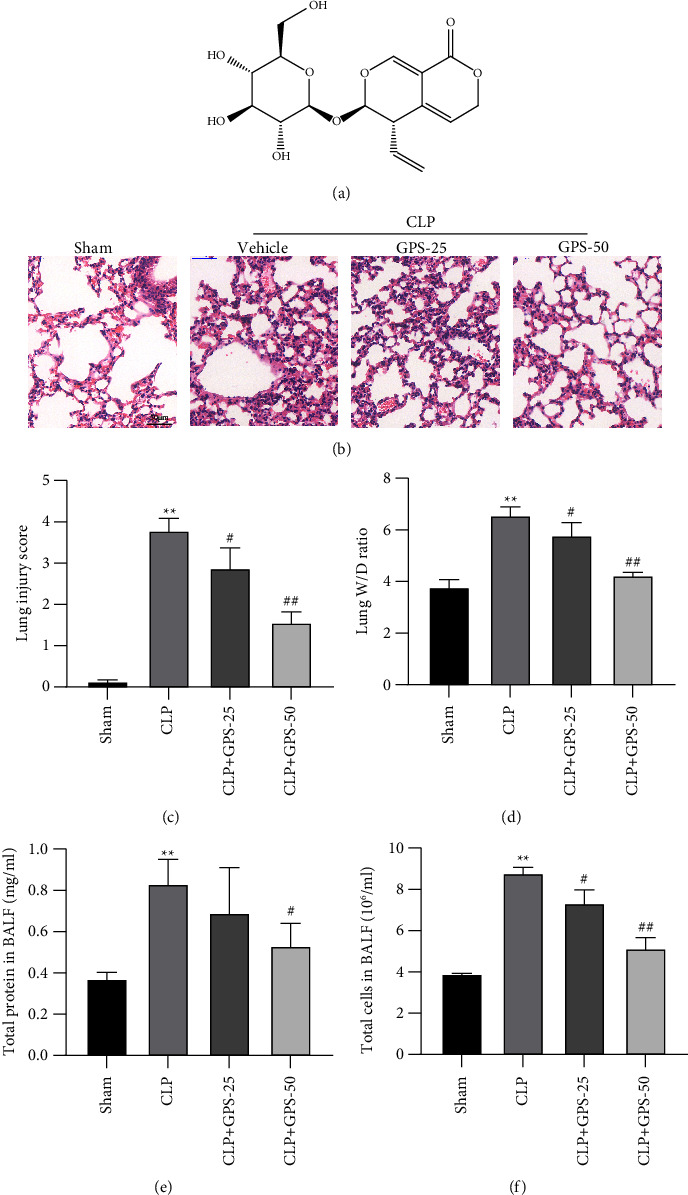
Various concentrations of GPS were injected into rats to alleviate the ALI caused by sepsis. (a) Chemical structure of GPS. (b) HE was performed to evaluate the histological changes of the collected lung tissues, scale bar = 50 *μ*m. (c) Lung injury score. (d) The wet/dry ratio of the right lung was detected. (e, f) Total protein content and total number of cells in BALF were measured via BCA assay kit and blood counting chamber. ^∗^A comparison with the Sham group. ^#^A comparison with the CLP group. ^∗∗^*p* < 0.01, ^#^*p* < 0.05, and ^##^*p* < 0.01.

The chemical composition of GPS is C16H20O9 (Figure 1(a)). Compared to the CLP group, the degree of inflammation in the lungs of rats in the CLP + GPS-25 and CLP + GPS-50 groups was reduced (Figure 1(b)). In addition, the lung damage rate, W/D ratio, total protein content, and total number of cells in BALF were significantly higher in the CLP group than in the Sham group (*P* < 0.05) (Figures 1(c), 1(d), 1(e), and 1(f)). This indicates that we have successfully established a rat model of CLP. The lung damage rate and W/D ratio were lower in the CLP + GPS-25 and CLP + GPS-50 groups than in the CLP group (*P* < 0.05) (Figures 1(c) and 1(d)). When compared to the CLP group, both the CLP + GPS-25 and CLP + GPS-50 groups exhibited reduced levels of total protein content and total number of cells (*P* < 0.05) (Figures 1(e) and 1(f)). According to these findings, injection of GPS improved the symptoms of ALI caused by sepsis in rats.

### 4.2. GPS Repress the Inflammatory Responses in the Rats with Sepsis-Induced ALI

**Figure 2:**
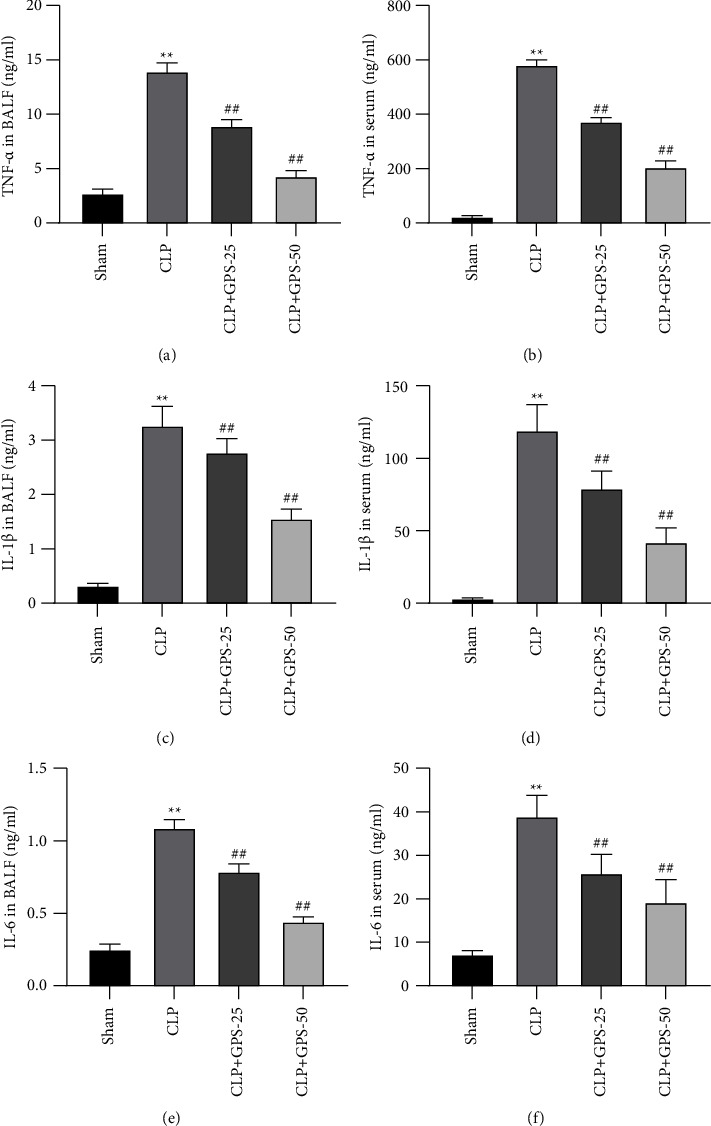
Injecting rats with GPS of different concentrations significantly inhibited the inflammatory response induced by sepsis. (a–f) The levels of inflammatory cytokines (IL-1*β*, IL-6, and TNF-*α*) in the BALF and serum were tested using the appropriate ELISA kits. ^∗^A comparison with the Sham group. ^#^A comparison with the CLP group. ^∗∗^*p*  <  0.01 and ^##^*p*  <  0.01.

The levels of the proinflammatory factors (TNF-*α*, IL-1*β*, and IL-6) in the serum and BALF of the CLP group were considerably greater than that of the Sham group (*P* < 0.01) (Figures 2(a), 2(b), 2(c), 2(d), 2(e), and 2(f)). However, GPS can reduce the levels of the proinflammatory factors (TNF-*α*, IL-1*β*, and IL-6) in the serum and BALF (*P* < 0.01) (Figures 2(a), 2(b), 2(c), 2(d), 2(e), and 2(f)). Together, GPS might protect the rats against the effects of inflammation caused by sepsis.

### 4.3. GPS Attenuates Sepsis-Induced Nitrosative Stress in the Lung Tissues of Rats

**Figure 3:**
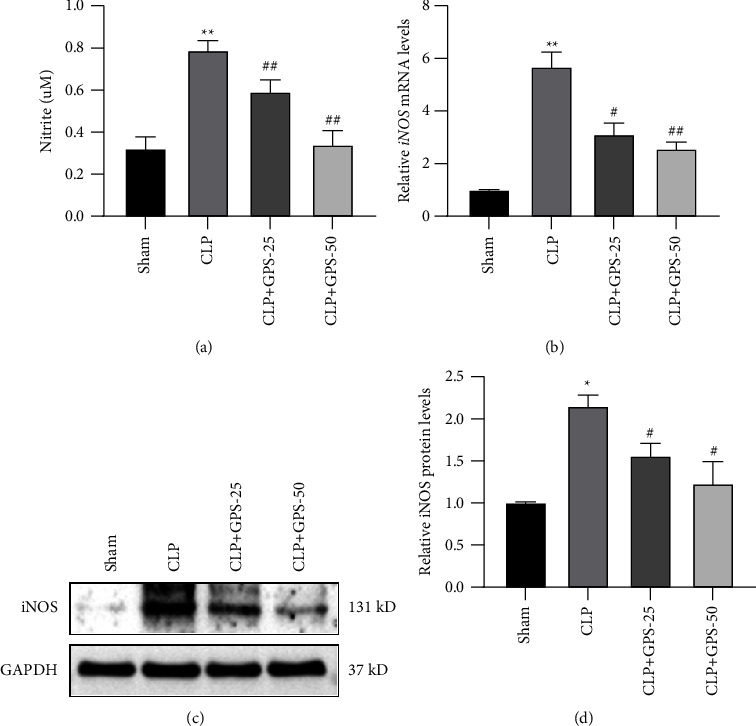
Injecting different concentrations of GPS alleviated the nitrogen stress caused by sepsis in rat lung tissues. (a) The concentration of nitrite in the lung tissues was tested using the Griess reagent. (b–d) The mRNA and protein levels of iNOS in the lung tissues were confirmed via RT-qPCR and western blot, respectively. ^∗^A comparison with the Sham group. ^#^A comparison with the CLP group. ^∗^*p* < 0.05, ^∗∗^*p* < 0.01, ^#^*p* < 0.05, and ^##^*p* < 0.01.

The Griess reagent was used to determine the level of nitrite present in the lung tissues. The findings indicated that a lower quantity of nitrite was seen when the GPS concentration increased (*P* < 0.01) (Figure 3(a)). Meanwhile, the mRNA and protein expressions of iNOS were decreased in the lung tissues of the CLP + GPS-25 and CLP + GPS-50 groups (*P* < 0.05) (Figures 3(b), 3(c), and 3(d)). Based on these findings, it seems that GPS reduces the amount of nitrosative stress due to sepsis in the lung tissues of rats.

### 4.4. GPS Alleviates Sepsis-Induced Oxidative Stress in the Lung Tissues of Rats

**Figure 4:**
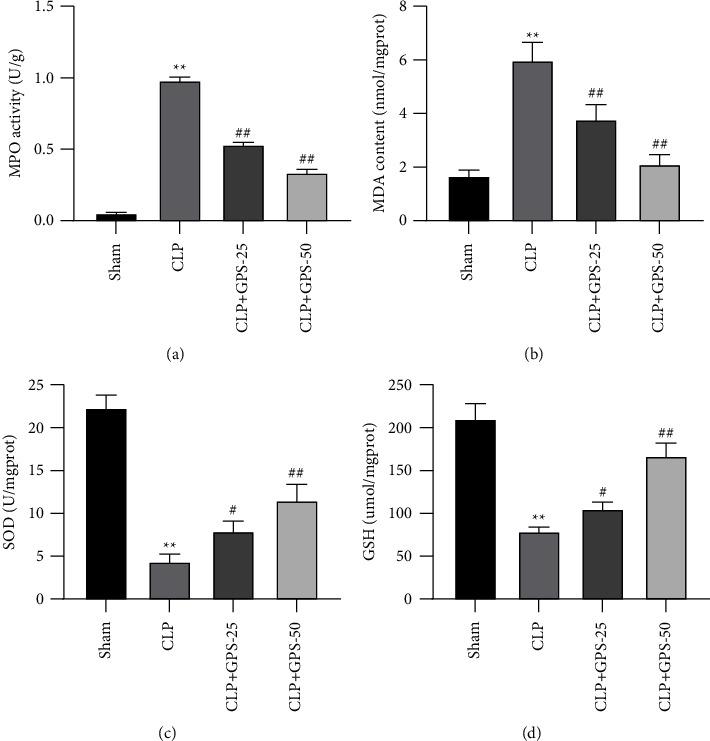
Injecting different concentrations of GPS alleviated sepsis-induced oxidative stress in the lung tissues of rats. (a) The MPO activity in the lung tissues was measured using a myeloperoxidase assay kit. (b–d) The contents of MDA, SOD, and GSH in the lung tissues were tested using their corresponding kits. ^∗^A comparison with the Sham group. ^#^A comparison with the CLP group. ^∗^*p* < 0.05, ^∗∗^*p* < 0.01, ^#^*p* < 0.05, and ^##^*p* < 0.01.

It was discovered that the MPO and MDA levels were considerably higher in the CLP group than in the Sham group, but the SOD and GSH levels were significantly lower (*P* < 0.01) (Figures 4(a), 4(b), 4(c), and 4(d)). When compared to the CLP group, the MPO and MDA levels were lower in the CLP + GPS-25 and CLP + GPS-50 groups. Contrarily, the SOD and GSH levels were greater in the CLP + GPS-25 and CLP + GPS-50 groups (*P* < 0.01) (Figures 4(a), 4(b), 4(c), and 4(d)). According to these data, GPS mitigated the oxidative stress due to sepsis in the lung tissues of rats.

### 4.5. GPS Protects the Lung Tissues from Sepsis-Induced Cell Apoptosis and Inflammation

**Figure 5:**
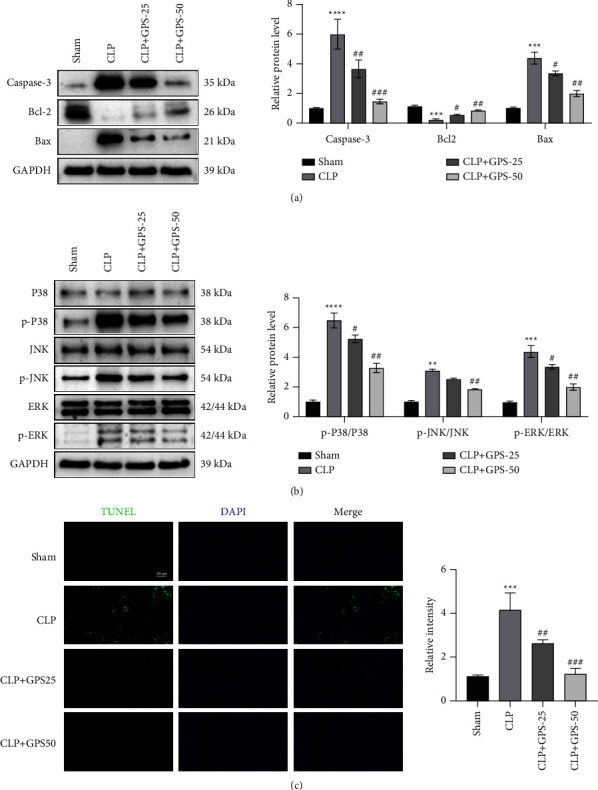
Adding GPS protects the lung tissues from damage caused by apoptosis and inflammation induced by sepsis *in vitro*. (a) Cell apoptosis-associated Caspase-3, Bcl-2, and Bax expressions in the lung tissues were observed via western blot. (b) Inflammation-related p-p38, p-ERK, and p-JNK expressions in the lung tissues were detected via western blot. (c) TUNEL assay showed cell apoptosis in the lung tissues, scale bar = 200 *μ*m. ^∗∗^*p* < 0.01, ^∗∗∗^*p* < 0.001, ^∗∗∗∗^*p* < 0.0001, ^#^*p* < 0.05, ^##^*p* < 0.01, and ^###^*p* < 0.001.

Cell apoptosis-associated protein expression in the lung tissues was evaluated via western blot. Compared with the Sham group, the Caspase-3 and Bax levels were increased, whereas the levels of Bcl-2 was decreased in the CLP group (Figure 5(a)). However, these phenotypes can be reversed by adding GLP (Figure 5(a)). Then, we detected the expression of inflammatory-related proteins in the lung tissues by western blot. Compared with the Sham group, the p-p38, p-ERK, and p-JNK levels in the CLP group were increased (Figure 5(b)). Compared with the CLP group, the p-p38, p-ERK, and p-JNK levels in the CLP + GPS-25 and CLP + GPS-50 groups were decreased (Figure 5(b)). The TUNEL assay results showed that GPS protects the lung tissues from sepsis-induced cell apoptosis (Figure 5(c)). These results suggest that GPS protects the lung tissues from sepsis-induced cell apoptosis and inflammation.

### 4.6. GPS Protects BEAS-2B Cells from LPS-Induced Cell Apoptosis and Inflammation

**Figure 6:**
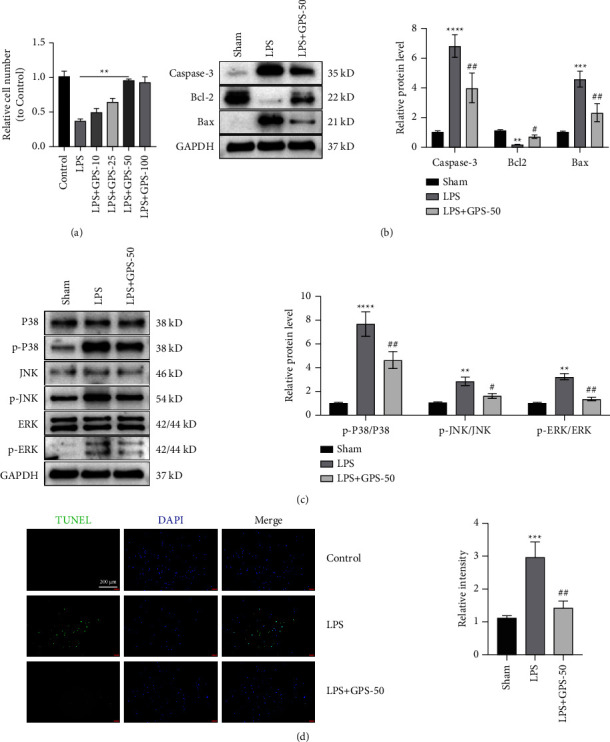
GPS protects BEAS-2B cells from the LPS-induced cell apoptosis and inflammation. (a) The relative cell number to control after adding GPS based on the CCK-8 assay results. (b) Cell apoptosis-associated Caspase-3, Bcl-2, and Bax expressions in the BEAS-2B cells were evaluated via western blot. (c) Inflammation-related p-p38, p-ERK, and p-JNK expressions in the BEAS-2B cells were detected via western blot. (d) TUNEL assay showed cell apoptosis in BEAS-2B cells, scale bar = 200 *μ*m. ^∗∗^*p* < 0.01, ^∗∗∗^*p* < 0.001, ^∗∗∗∗^*p* < 0.0001, ^#^*p* < 0.05, and ^##^*p* < 0.01.

The CCK-8 assay results showed that GPS concentrations >50 *μ*M had a protective effect on LPS-induced ALI (*P* < 0.01) (Figure 6(a)); hence, this GPS concentration was selected for subsequent studies. Compared with the LPS group, GPS can obviously reduce the Caspase-3 and Bax levels and increase the Bcl-2 level (Figure 6(b)). LPS significantly increased the expression of p-p38, p-ERK, and p-JNK in BEAS-2B cells; as compared with the control group, pretreatment with GPS dramatically decreased the expression of p-p38, p-ERK, and p-JNK induced by LPS (Figure 6(c)). The TUNEL assay results showed that GPS can alleviate the LPS-induced cell apoptosis (Figure 6(d)). These results suggest that GPS can decrease the LPS-induced cell apoptosis and inflammation.

## 5. Discussion

Sepsis is associated with high mortality and incidence rates [20]. Patients admitted in critical care units are more likely to die of sepsis than any other. Owing to multiorgan damage, the mortality rate might reach as high as 30%–70% [21]. The first sign of organ damage during the progression of sepsis is observed in the lungs, followed by the presence of lesions to other organs in sequential order [22, 23]. ALI/acute respiratory distress syndrome (ARDS), which is caused by sepsis, has the highest incidence and fatality rates among other conditions in the intensive care unit [24]. In many cases, the uncontrolled inflammatory reaction is due to the release of inflammatory mediators by the activated inflammatory cells, which then act together on alveolar epithelial cells and alveolar capillary endothelial cells. This results in a decrease in the function of the pulmonary blood barrier, an increase in capillary basement membrane permeability, and a considerable amount of liquid that is rich in protein seeping out of blood vessels and into the alveolar space, subsequently leading to the simultaneous development of alveolar and interstitial pulmonary edema and the production of hyaline membrane in the alveoli. The primary pathophysiological characteristics were an imbalance in ventilation and blood flow, reduced lung compliance, and lower lung capacity. The clinical symptoms include increased acute respiratory distress, hypoxemia that does not respond to treatment, and noncardiogenic pulmonary edema [25–27]. It is now thought that the mechanical stress induced by excessive mechanical ventilation may contribute to lung inflammation [28]. Lung inflammation is a considerable iatrogenic cause of ALI that might occur in clinical practice [29]. Sumatriptan reduces the mortality rate of C57BL/6 mice with septicemia due to its anti-inflammatory and antioxidative stress effects. Therefore, it is of considerable clinical value to better understand the relevance of sepsis and examine optimal therapeutic strategies other than mechanical ventilation for patients with this clinical condition, which is associated with a high mortality rate. In this study, we were able to observe the effects of GPS therapy, which effectively reduced the levels of TNF-*α*, IL-1*β*, IL-6, nitrogen stress, oxidative stress, and the severity of ALI at both the whole animal and molecular levels. Furthermore, GPS alleviate LPS-induced ALI by regulating the inflammatory response and cell apoptosis in BEAS-2B. Hence, this research lays a foundation for the potential use of GPS in the treatment of sepsis-induced ALI.

The CLP-induced sepsis model is most closely representing the pathophysiology and clinical signs of sepsis [30]. This is because CLP mimics the inflammatory response that occurs during sepsis. In the present study, we used rats with CLP-induced sepsis to evaluate the preventative effect of GPS and its mechanism among sepsis-induced ALI cases. The rats were supplemented with GPS throughout the course of the study. Our study data revealed that the GPS may reduce the severity of the pathological damage caused by CLP-induced sepsis in rats. Moreover, GPS reduced the pathological score, lung wet-to-dry ratio, and TNF-*α,* IL-1*β*, and IL-6 levels in BALF. Contrarily, the levels of MPO and MDA in the lung tissue were increased by GPS, while simultaneously reducing the levels of SOD and GSH. Pulmonary interstitial edema and alveolar injury are the manifestations of lung disease that are most noticeable to the naked eye. The ratio of the amount of water in the lungs to the amount of water that is normally found in the lungs is regarded as a direct measure of pulmonary edema as well as a common indication of pulmonary inflammation. According to our study findings, the administration of GPS therapy to rats with CLP-induced sepsis resulted in a reduction in the percentage of the lung tissue that was wet in comparison to that which was dry. GPS was able to reduce the severity of the complications of the sepsis generated by CLP in rats with lung injury.

Inflammation is the most important component in determining the prognosis of patients with sepsis in its early stages [31]. Exudation and aggregation of pulmonary neutrophils have historically been believed to form a crucial cytological link in terms of the etiology of ALI [32]. Both processes are associated with the condition. TNF-*α* activated macrophages can release MIP-2, which is a powerful neutrophil chemotactic factor. MIP-2 is secreted after the macrophages have been stimulated. Macrophages are responsible for the production of a chemical known as MIP-2. Neutrophils are among the cell types that are especially targeted by this toxin. Chemical chemotaxis and activation are the two ways in which MIP-2 contributes to the inflammatory response. Chemical chemotaxis is the third mechanism. Owing to its position as a substantial proinflammatory factor, IL-18 can induce the production and release of other inflammatory mediators (such as TNF-*α*), which may lead to inflammation that is not under control. This may be the case if the inflammation is allowed to continue. The potential of TNF-*α* to induce an inflammatory cascade response, increase the formation of free radicals, and worsen the degree of lung injury is among its primary characteristics [33, 34]. According to the study findings, GPS was able to reduce the inflammatory response in rats that had CLP-induced sepsis by reducing the TNF-, IL-1, and IL-6 levels in their BALF. Based on these findings, GPS may be able to prevent the generation of cytokines that contribute to inflammation. However, further studies are necessary to understand the process behind the more specific and all-encompassing molecular approach.

## 6. Conclusion

GPS can significantly suppress the inflammatory response, nitric oxide stress, oxidative stress, and changes in acute lung injury after sepsis-induced ALI at the whole animal and molecular levels. This is an interesting new concept for research into the prevention and therapeutic treatment of acute lung damage caused by sepsis.

## Data Availability

The datasets generated during and/or analyzed during the current study are available from the corresponding author on reasonable request.
